# Deciphering pathogenicity and antibiotic resistance islands in methicillin-resistant *Staphylococcus aureus* genomes

**DOI:** 10.1098/rsob.170094

**Published:** 2017-12-20

**Authors:** Mehul Jani, Soham Sengupta, Kelsey Hu, Rajeev K. Azad

**Affiliations:** 1 Department of Biological Sciences, University of North Texas, Denton, TX 76203, USA; 2 Texas Academy of Mathematics and Science, University of North Texas, Denton, TX 76203, USA; 3 Department of Mathematics, University of North Texas, Denton, TX 76203, USA

**Keywords:** *Staphylococcus aureus*, antibiotic resistance, genomic islands, MRSA, pathogenicity

## Abstract

*Staphylococcus aureus* is a versatile pathogen that is capable of causing infections in both humans and animals. It can cause furuncles, septicaemia, pneumonia and endocarditis. Adaptation of *S. aureus* to the modern hospital environment has been facilitated, in part, by the horizontal acquisition of drug resistance genes, such as *mecA* gene that imparts resistance to methicillin. Horizontal acquisitions of islands of genes harbouring virulence and antibiotic resistance genes have made *S. aureus* resistant to commonly used antibiotics. To decipher genomic islands (GIs) in 22 hospital- and 9 community-associated methicillin-resistant *S. aureus* strains and classify a subset of GIs carrying virulence and resistance genes as pathogenicity and resistance islands respectively, we applied a host of methods for localizing genomic islands in prokaryotic genomes. Surprisingly, *none* of the frequently used GI prediction methods could perform well in delineating the resistance islands in the *S. aureus* genomes. Rather, a gene clustering procedure exploiting biases in codon usage for identifying horizontally transferred genes outperformed the current methods for GI detection, in particular in identifying the known islands in *S. aureus* including the SCC*mec* island that harbours the *mecA* resistance gene. The gene clustering approach also identified novel, as yet unreported islands, with many of these found to harbour virulence and/or resistance genes. These as yet unexplored islands may provide valuable information on the evolution of drug resistance in *S. aureus*.

## 1. Introduction

*Staphylococcus aureus* is a Gram-positive coccus and an important human pathogen responsible for nosocomial and community-acquired infections. It colonizes mucous membranes and skin, and can survive even in harsh environmental conditions. Earlier treatment options for *S. aureus* infections included penicillin G. However, an increase in the emergence of strains resistant to methicillin made treatment very difficult for *S. aureus* infections [1–6]. These methicillin-resistant *S. aureus* (MRSA) strains have since become resistant to other antibiotics including macrolides, lincosamides and all beta-lactams. More recently, multi-drug-resistant MRSA strains have acquired resistance against vancomycin [7,8].

Acquisitions of drug resistance factors are facilitated by horizontal transfer of plasmids, transposons and other mobile genetic elements. For example, the resistance to methicillin was gained by the acquisition of an ‘island’ of genes, namely the staphylococcal chromosome cassette methicillin-resistance (SCC*mec*) island. SCC*mec* carries the PBP2a-encoding *mecA* gene, which is responsible for methicillin resistance. Several studies have, therefore, focused on identifying these genomic islands (GIs) to understand the underlying mechanisms of the emergence of complex antibiotic resistance patterns mediated by horizontal gene transfer (HGT) [3]. These islands and the other mobile genetic elements such as phages, transposons and chromosomal cassettes together constitute the auxiliary or accessory genome of *S. aureus.* The core genome has previously been reported to be composed of approximately 95% or more of all *S. aureus* genes [9]; recent studies have revealed recombination hotspots for mobile elements even within the *S. aureus* core genome [10]. The *S. aureus* genome backbone is composed of genes present in all or most *S. aureus* strains, whereas the accessory genome harbours genes that are unique to a strain or are present in only a few strains and are likely a consequence of acquisitions from distantly related or unrelated organisms through HGT. Besides helping gain resistance to antibiotics, foreign genes have also aided *S. aureus* in causing infections and proliferating in a community setting. For example, the acquisition of Panton–Valentine leucocidin gene by MRSA has given rise to community-acquired MRSA (CA-MRSA) [1,2]. Although *S. aureus* has traditionally been classified as a nosocomial agent and was believed to cause only hospital-associated infections, the cases of CA-MRSA infection have been increasing owing to the emergence of new CA-MRSA strains as a consequence of HGT [7,11].

Quantifying resistance and virulence-associated GIs is central to understanding the emergence and evolution of hospital-associated (HA) and CA-MRSA strains. In our quest for a robust method for GI detection in MRSA among the currently available GI detection methods, we first assessed their ability to detect known islands in MRSA. The methods displayed varying levels of sensitivity in identifying the known GIs in MRSA, with none found satisfactory in localizing the SCC*mec* resistance island. We therefore explored an information-entropy-based gene clustering method that uses codon usage bias to identify genes originating from different sources [12]. Although a bottom-up approach (i.e. gene-by-gene analysis) and not usually recommended for detecting large acquisitions, it displayed remarkable success in localizing resistance and other known islands in MRSA in comparison with the existing methods. This is a significant development as more robust detection of MRSA GIs is a precursor to an effective downstream analysis for understanding the emergence and evolution of MRSA strains through GI acquisition. In what follows, we briefly describe the methods used in this study, discuss their performance on localization of known GIs in a representative set of HA- and CA-MRSA genomes, highlight our novel predictions, and conclude with remarks on the impact of this study and future directions.

## 2. Material and methods

### 2.1. Methicillin-resistant *Staphylococcus aureus* genomes

The complete genome sequences and gene coordinates for 22 HA- and 9 CA-MRSA strains were obtained from GenBank (https://www.ncbi.nlm.nih.gov/genbank/). We referred to the previous studies to compile a high confidence set of known MRSA GIs [5,13,14]. The coordinates of known MRSA GIs, their codon and GC features, and supporting evidence from the corresponding studies are given in table 1 and the electronic supplementary material, tables S1 and S2.

**Table 1.** Coordinates of known GIs in the genomes of the five MRSA strains and the corresponding JS-CB predicted GI coordinates. Coordinates of known GIs in MRSA strains [5,13,14] and coordinates of the corresponding JS-CB predicted GIs in the MRSA genomes are indicated in base pairs (bp). Δ, remnant GI [5].

	USA300		MW2		N315		Mu50		COL	
island	known [5,13]	predicted	known [5,14]	predicted	known [5,14]	predicted	known [5,14]	predicted	known [5,14]	predicted
SCC*mec*	34 173–57 914	34 513–113 460	34 150–58 278	34 490–87 067	34 158–87 119	33 692–111 612	34 158–87 085	34 455–107 910	34 173–68 085	34 375–51 600
ACME	57 915–88 900	34 513–113 460	—	—	—	—	—	—	—	—
νSaα	441 501–473 470	441 501–479 066	416 307–452 099	416 307–440 048	436 162–466 813	436 159–467 952	461 919–491 326	477 065–492 473	465 424–489 723	451 796–476 971
SaPI-5	881 835–895 807	843 347–898 530	—	—	—	—	—	—	—	—
φSa2	1 545 912–1 592 050	1 557 369–1 606 675	1 529 123–1 575 042	1 540 580–1 589 663	—	—	—	—	—	—
νSaβ	1 924 777–1 959 376	1 930 682–1 936 843	1 890 800–1 922 552	1 895 586–1 901 747	1 854 608–1 881 615	—	1 932 523–1 961 464	—	1 902 466–1 938 731	1 909 272–1 914 665
φSa3	2 084 658–2 127 720	2 096 778–2 138 923	2 046 205–2 088 820	2 058 798–2 100 838	2 005 321–2 049 591	2 018 761–2 072 272	2 083 238–2 126 304	2 095 697–2 148 702	—	—
νSa4	^Δ^2136710–2 139 851	2 096 778–2 138 923	^Δ^2097809–2 100 950	2 058 798–2 100 838	2 056 679–2 072 358	2 018 761–2 072 272	2 133 235–2 148 912	2 095 697–2 148 702	^Δ^2072899–2 076 041	2 063 728–2 075 113
νSaγ	1 149 668–1 169 542	—	1 133 469–1 153 549	—	1 132 235–1 153 775	—	1 208 629–1 230 219	—	1 173 206–1 193 358	—
φSa1	—	—	—	—	—	—	917 453–962 005	916 042–981 803	—	—
νSa3	—	—	839 358–853 808	827 901–856 739	—	—	868 373–882 872	868 462–886 899	—	—
φCOL	—	—	—	—	—	—	—	—	354 674–398 267	315 294–377 918
νSa1	—	—	—	—	—	—	—	—	903 332–919 283	873 009–922 004

### 2.2. Genomic island detection methods

We used the following GI prediction methods at their default parameter setting unless mentioned otherwise (see §3.2).

#### 2.2.1. Alien_Hunter

Interpolated variable order motifs (IVOM) [15] or Alien_Hunter uses an interpolated Markov model accounting for variable order motifs to assess the compositional difference between a region within a moving window and the genome. Compositionally atypical regions are identified as GIs.

#### 2.2.2. PredictBias

PredictBias examines genomic regions within a moving window and annotates successive ORFs with atypical codon usage bias and either atypical GC composition or dinucleotide composition as GIs [16]. PredictBias also examines the presence of virulence-associated genes within clusters of eight contiguous ORFs; if four of these genes have significant BLAST hits in the virulence factor database (VFDB), the cluster is an annotated GI even if it does not display atypicality in dinucleotide or codon usage bias.

#### 2.2.3. SeqWord

SeqWord uses oligonucleotide usage (OU) patterns to assess compositional differences in the genome [17]. Genomic islands are identified as compositionally divergent regions based on local and global OU patterns.

#### 2.2.4. IslandViewer

This integrated visualization tool provides predictions from three programs, IslandPath-DIMOB, SIGI-HMM, and IslandPick along with the visualization of GIs.

#### 2.2.5. Zisland Explorer

This program uses cumulative GC profile to segment the genome first, assesses the GC heterogeneity to exclude the core (vertically inherited) segments, and finally uses the codon usage bias to identify putative GIs [18].

#### 2.2.6. GIHunter

Genomic Island Hunter (GIHunter) [19] uses a decision tree to identify GIs. It builds a GI/non-GI gene classifier using the dataset of known GIs and non-GIs. GI features, such as sequence composition, presence of mobility genes and integration sites, are used as classification features.

#### 2.2.7. MSGIP

Mean Shift Genomic Island Predictor (MSGIP) is a clustering method based on mean shift algorithm, a non-parametric method that calculates mean shift vector and moves the density estimation window in the direction of local density maxima, until the convergence is reached [20]. Following clustering, MSGIP identifies GIs as clusters of atypical windows with length not exceeding 200 kbp.

#### 2.2.8. GEMINI

GEMINI is a genome-mining tool based on a recursive segmentation and clustering procedure [21]. GEMINI segments a genome recursively into compositionally homogeneous segments within a statistical hypothesis testing framework and then groups similar segments within the same framework. Additionally, GEMINI exploits segment context information to achieve more robust clustering. Potentially, vertically inherited or native segments representing the genome backbone are identified by the largest cluster harbouring segments of ‘typical’ composition. Segments of ‘atypical’ composition, representing putative horizontally acquired DNAs, are assigned to the numerous smaller clusters each representing a likely donor source. Large atypical segments, 8 kbp or more in size, are predicted as GIs.

#### 2.2.9. Gene clustering (JS-CB)

JS-CB is a gene clustering method for identifying putative horizontally acquired genes [12]. Although not designed specifically to detect GIs, we tested its ability to identify GI-borne genes in MRSA genomes [12]. Briefly, JS-CB uses Jensen–Shannon (JS) divergence measure [22,23] to assess the difference in codon usage bias between two genes. Genes with similar codon usage bias are grouped together using an agglomerative hierarchical clustering. JS-CB begins with all individual genes as single-gene clusters, followed by pairwise comparison of the clusters. The two most similar gene clusters (in terms of JS divergence) are merged iteratively within a statistical hypothesis framework. If the *p*-value, computed based on an analytic approximation of the probability distribution of JS divergence, is less than a preset significance level (default: 0.005), the gene classes are deemed different, otherwise they are merged. The process is performed recursively resulting in clusters of genes with similar codon usage bias. The largest cluster represents the native genes, while the numerous smaller clusters harbour putative alien genes, each representing a potential donor. The clusters of highly expressed genes, e.g. the ribosomal protein genes, are identified and merged with the native cluster. Eight or more contiguous alien genes are annotated GIs.

### 2.3. Assessment of genomic island prediction methods

To assess the performance of GI prediction tools, we obtained the recall (sensitivity), precision, F-measure (harmonic mean of recall and precision), and performance coefficient (PC) in identifying GIs for each method, as defined below.
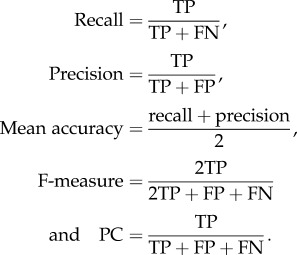
Here, TP ≡ true positive, FN ≡ false negative, FP ≡ false positive.

A predicted GI is deemed a match or true positive if it overlaps with a known GI (see also §3.1 for further details), otherwise it is called a false positive. A misclassified known GI is annotated false negative.

## 3. Results and discussion

### 3.1. Comparison of genomic island prediction tools

**Figure 1.**
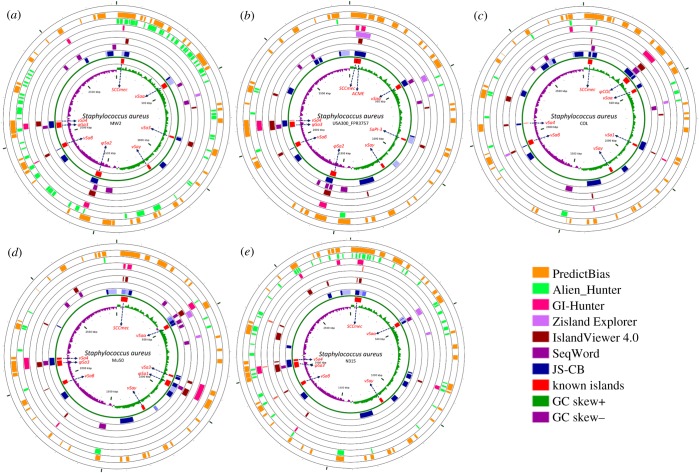
GIs predicted in five *Staphylococcus aureus* MRSA strains by different methods. From the outside inward: first seven circles show the GIs predicted by MSGIP (blank as no island identified), PredictBias, Alien_Hunter, GIHunter, Zisland Explorer, IslandViewer, SeqWord and JS-CB; known islands are shown in the eighth circle with arrows pointing to their names. Mosaic GIs predicted by JS-CB have their distinct segments shown in different shades of blue colour. Ninth circle represents GC skew in green and violet colour. The reference *S. aureus* strains used were (*a*) MW2, (*b*) USA300_FPR3757, (*c*) COL, (*d*) Mu50 and (*e*) N315. The figure was generated using CGviewer 3.0 [27].

To understand the contributions of GIs in the evolution of drug-resistant MRSA, we first assessed the published GI detection methods for their ability to identify the well-characterized GIs in the MRSA genomes. These methods were applied to a representative set of MRSA genomes that included three HA-MRSA and two CA-MRSA genomes with known GIs (table 2). Results from the application of current methods, namely JS-CB (gene clustering based on codon usage bias) [12], GIHunter (DGI-database of GIs of 2000 bacterial genomes) [19], IslandPick (automated comparative genomics approach) [24], Zisland Explorer (based on a segmentation algorithm) [18], IslandViewer (database of predicted GIs from three methods) [25], PredictBias (based on G + C content, dinucleotide composition, codon usage bias, and the presence of virulence genes) [16], SeqWord (based on oligonucleotide usage) [17], Alien_Hunter (based on an interpolated Markov model accounting for variable length *k*-mers and a hidden Markov model) [15], SIGI-HMM (based on a hidden Markov model of codon usage) [26] and MSGIP (clustering using a mean shift algorithm) [20], to MRSA strains MW2, USA300_ FPR3757, COL, Mu50 and N315 are shown in figure 1*a*–*e*. The known GIs are shown in red on the innermost track in figure 1*a*–*e*. The number of predicted GIs overlapping the known GIs, with overlap spanning over half of the known GI and the predicted GI not greater than twice the size of the known GI, is listed in the electronic supplementary material, table S3 for each method. PredictBias identified 27 GIs and JS-CB detected 23 GIs, while GIHunter, SeqWord, Zisland Explorer, IslandViewer and Alien_Hunter identified much fewer known GIs. MSGIP did not identify any known GIs. Although PredictBias identified more islands compared to JS-CB, this was achieved at the expense of potentially many false positives (PredictBias predicted 260 GIs, while JS-CB predicted only 66). We quantified a method's ability to identify maximum known GIs with fewer predictions by computing the recall and precision in identifying known islands in the five selected MRSA strains (table 2). If a method generated numerous segments spanning parts of a known GI, the largest segment, i.e. the segment with largest overlap with the GI, was considered the predicted GI corresponding to the known GI. If a prediction overlapped more than one known GI, then each overlap of the predicted segment with the known GIs was considered. In addition to the 50% overlap cut-off for GI identification, the recall and precision were also obtained at cut-offs 75% and 95% (table 2, cut-off *N*% means that at least *N*% of a known GI needs to be identified as an alien segment for the prediction to be deemed a success). We thus assessed the ability of a method in identifying GIs as singular units that are mobilized across genomes in single evolutionary events through HGT. The mean values of the performance metrics (averaged over the five strains) are given in table 3. At the cut-off of 50%, JS-CB outperformed the next best performing tools PredictBias, GIHunter and IslandViewer by at least approximately 4% each in the mean accuracy. At the cut-off of 75%, JS-CB performed marginally better than the next best performing tools. At the stringent cut-off of 95%, JS-CB outperformed PredictBias, GIHunter and IslandViewer by approximately 10%, approximately 17%, and approximately 5% respectively in the mean accuracy. We observed similar trend with F-measure and performance coefficient (table 3). Apparently, JS-CB balances recall and precision better than any other methods resulting in overall higher accuracy (tables 2 and 3). JS-CB and PredictBias performed much better in identifying the known GIs than the other methods, as indicated by higher recall values; however, PredictBias predicts substantially more GIs than JS-CB and hence suffers from the low precision values (tables 2 and 3).

**Table 2.** Assessment of GI prediction tools: Recall and precision in identifying known GIs in five MRSA strains are shown for GI prediction methods. Highest values of recall and precision among all methods are shown in red; recall *N*%: (number of known GIs with at least *N*% of nucleotides classified correctly)/(number of known GIs); precision *N*%: (number of known GIs with at least *N*% of nucleotides classified correctly)/(number of GIs predicted). JS-CB, Jensen–Shannon Codon-Bias; MSGIP, Mean Shift Genomic Island Predictor.

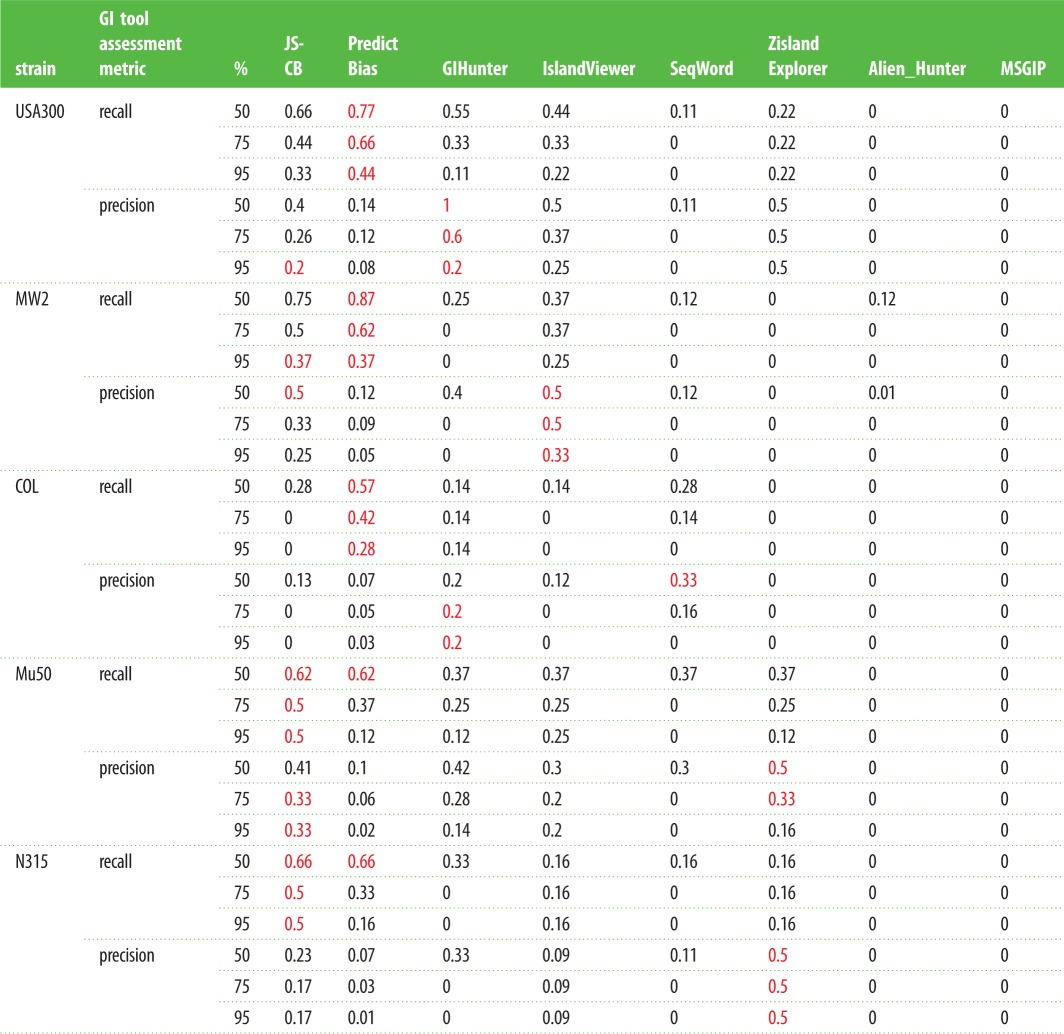

**Table 3.** Performance metrics of GI prediction tools: recall, precision, mean accuracy, F-measure, and performance coefficient of GI prediction methods averaged over the five MRSA strains are shown for different overlap cut-offs, with highest values among all methods shown in red; recall *N*%: (number of known GIs with at least *N*% of nucleotides classified correctly)/(number of known GIs); precision *N*%: (number of known GIs with at least *N*% of nucleotides classified correctly)/(number of GIs predicted). JS-CB, Jensen–Shannon Codon-Bias.

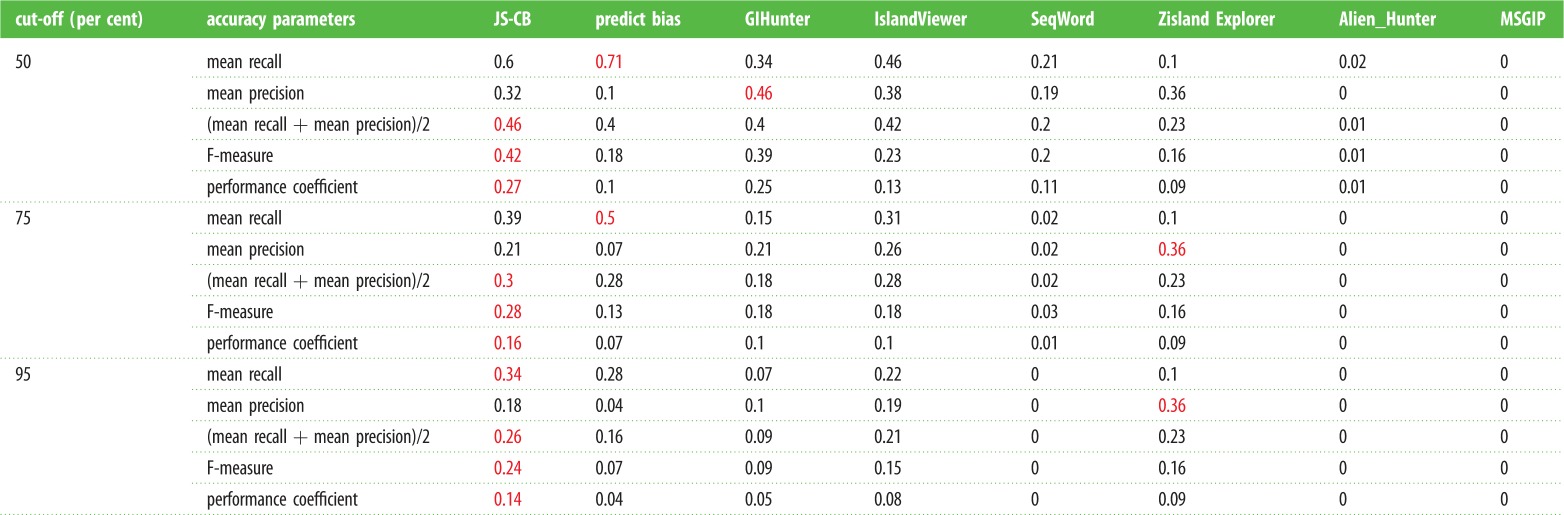

As many methods often over-segment (i.e. predict more than one segment spanning parts of a GI) or under-segment (i.e. predict a segment that spans whole or parts of more than one GI), we also evaluated their performance through a different assessment criterion—now considering all spanning segments not just the segment having the largest overlap with the GI for the former, and similarly considering all GIs that are spanned wholly or partially by a predicted segment with the restriction that the size of the predicted segment is not more than twice the GI size now removed for the latter. This allowed a known GI to be deemed identified by a method at *N*% cut-off if the overlaps together exceeded *N*% threshold for the former, and similarly multiple known GIs were deemed identified if the predicted segment overlapped each of these GIs by over *N*% of their size. While this raised the recall of these methods, the over-segmentation (identifying a GI as many fragments) was penalized by considering all predicted segments and the under-segmentation (identifying many GIs as one segment) by considering both GI and non-GI regions spanned by the predicted segment in computing the precision of the methods (Case A in the electronic supplementary material, table S4). Alternatively, for under-segmentation, only one GI with the highest per cent overlap among all GIs spanned by a predicted segment was considered in obtaining the recall and precision of a method (Case B in the electronic supplementary material, table S4). This is a reasonable approach that ensures that over-segmenting or under-segmenting methods never reach perfect accuracy (100% recall and 100% precision) as one would expect them not to. JS-CB outperformed other methods for these evaluation criteria. JS-CB yielded the best overall performance, with highest mean accuracy, F-measure, and performance coefficient for all five reference strains at all three cut-offs. The next best performing methods were GIHunter (at 50% cut-off), IslandViewer (at 75% cut-off) and Zisland Explorer (at 95% cut-off) (electronic supplementary material, table S4). Because the complete set of actual GIs in any strain is yet to be determined, these results should be interpreted with caution; in fact, some of the ‘false positives’ could indeed be true positives. However, the relative performance of the methods could still be assessed based on their ability to identify the already-known GIs with fewer predictions; a method balancing this trade-off well, reflected in terms of highest accuracy among the compared methods, could be deemed most successful among the methods.

We performed an additional assessment of the methods by constructing artificial MRSA genomes as described below. The artificial genomes enabled a more objective assessment as the evolutionary history of the segments in these genomes is already known. To construct an artificial recipient MRSA genome, we selected the *S. aureus* subsp. aureus MW2 genome and purged it of all known GIs as well as sequences that were predicted GIs by any of the nine methods considered in this study. We thus obtained the backbone *S. aureus* MW2 genome that was identified as core genome by all methods. Artificial donor genomes were similarly constructed, using the genomes of *Alkaliphilus metalliredigens* QYMF (NC_009633.1), *Erysipelothrix rhusiopathiae str*. Fujisawa (NC_015601.1), *Macrococcus caseolyticus* JCSC5402 (NC_011999.1) and *Salinicoccus halodurans* H3B36 (CP011366.1), which were chosen to represent a broad range of phylogenetically related donors. *M. caseolyticus* and *S. halodurans* represent the genera *Macrococcus* and *Salinicoccus* respectively within the family Staphylococcaceae that *S. aureus* belongs to. *A. metalliredigens* and *E. rhusiopathiae* represent the classes Clostridia and Erysipelotrichi respectively within the phylum Firmicutes that *S. aureus* belongs to. We then simulated the transfer of islands into this conservative core of the MW2 genome. Six DNA segments, between 40 and 50 kbp in size, were sampled from the donor genomes and inserted into the artificial MW2 genome, yielding approximately 82% native and approximately 18% alien composition. Ten random trials of this experiment yielded ten artificial genomes on which to assess the methods. Among the assessed methods, JS-CB yielded the highest values for all performance metrics, e.g. the values of F-measure (averaged over 10 artificial genomes) for JS-CB were 0.85, 0.81 and 0.55 at 50%, 75% and 95% cut-offs, whereas those for the next best performing methods were 0.4, 0.31 and 0.29 (Alien_Hunter), 0.37, 0.27 and 0.25 (SeqWord), and 0.29, 0.27 and 0.23 (IslandViewer), respectively (electronic supplementary material, table S5). Similarly, JS-CB outperformed other methods when the methods were evaluated through a different assessment criterion as discussed above (for both Case A and Case B, electronic supplementary material, table S6). The next best performing methods were IslandViewer, Alien_Hunter and SeqWord. We were unable to run PredictBias and GIHunter on artificial genomes; GIHunter returned errors for both artificial and the five reference strains (GI predictions for the reference strains were earlier retrieved from GIHunter's pre-computed database), and PredictBias could not recognize the GenBank annotation files of the artificial genomes). It should be noted that methods such as JS-CB have previously been assessed on simulated genomes of varying complexity [12]. GIs predicted by each method are shown in figure 1, and the performance of the methods on each representative MRSA genome and their ability to identify SCC*mec* are summarized below.

#### 3.1.1. *Staphylococcus aureus* strain MW2

Both Alien_Hunter and PredictBias predicted over 50 islands (including both pathogenicity islands and other GIs). By contrast, IslandViewer and GIHunter predicted only five islands in this strain. SCC*mec* was identified by JS-CB, IslandViewer, Alien_Hunter and PredictBias, but was completely missed by Zisland Explorer and SeqWord. JS-CB predicted 13 GIs identifying seven out of the eight known islands (SCC*mec*A, νSaα, νSa3, φSa2, νSaβ, φSa3 and νSa4). Of the seven known islands that overlapped with JS-CB predictions, six could be identified at the 50% cut-off, whereas at this cut-off GIHunter could identify only two known islands, and IslandViewer and Alien_Hunter could detect only one known island. Although PredictBias has the highest recall in identifying the known islands, JS-CB outperformed other methods in terms of precision as is also obvious from the comparison of coordinates of known and predicted islands (table 1 and figure 1).

#### 3.1.2*.* *Staphylococcus aureus* strain USA300_FPR3757

The number of predicted islands varied between three (Zisland Explorer) and 50 (PredictBias). SCC*mec* was identified by JS-CB, Zisland Explorer, GIHunter and PredictBias at the 50% cut-off. JS-CB predicted 15 islands, overlapping with eight of the nine known islands (SCC*mec*, ACME, νSaα, SaPI-5, φSa2, νSaβ, φSa3 and νSa4). At the 50% overlap cut-off, JS-CB was most successful, identifying seven of the nine known GIs. PredictBias and GIHunter were the next best, each identifying five of the nine known GIs.

#### 3.1.3. *Staphylococcus aureus* strain COL

Of the seven known islands, JS-CB's predictions overlapped with six (SCC*mec*, νSaα, νSaβ, νSa4 (remnant), φCOL, νSa1); however, only four (SCC*mec*, νSa4, φCOL, νSa1) had over 50% overlap with the predictions. JS-CB predicted an additional 11 GIs in this strain. SCC*mec* island was also identified by PredictBias and SeqWord. At the 50% overlap cut-off, PredictBias had higher recall than JS-CB but at the cost of many false positives; Zisland Explorer and MGSIP failed to identify any known islands at this cut-off.

#### 3.1.4. *Staphylococcus aureus* strain Mu50

JS-CB could identify six of the eight known GIs in *Staphylococcus aureus* strain Mu50, with five overlapping the respective predicted GIs by over 50%. SCC*mec* was identified by JS-CB and PredictBias at the 50% cut-off. At the 50% cut-off, Alien_Hunter and MGSIP failed to identify any known GIs in this strain.

#### 3.1.5. *Staphylococcus aureus* strain N315

Of the six known islands in this strain, JS-CB identified four islands at the 50% cut-off. SCC*mec* was identified by JS-CB, IslandViewer, GIHunter, Alien_Hunter and PredictBias. Zisland Explorer was able to detect only one of the known islands. Alien_Hunter could not identify any GI at the 50% cut-off. MGSIP failed to localize any of the known islands.

Our analysis shows that JS-CB has the highest recall for Mu50 and N315 strains at all cut-offs. PredictBias has highest recall for USA300, MW2 and COL (table 2). JS-CB outperforms all other GI prediction methods by approximately 5% or more in mean recall at the 95% cut-offs, and is outperformed by PredictBias by approximately 11% in mean recall at the lower cut-offs (table 3). PredictBias incurs false positives at much higher rate (mean precision ranges from approximately 4% to 10%, table 3) than JS-CB (mean precision ranges from approximately 18% to 32%, table 3). IslandViewer displays lower recall but higher precision in comparison with JS-CB. GIHunter and Seqword have much lower recall, with a mean recall of 34% and 21% respectively at the cut-off of 50% (table 3); If an island lacks mobility genes or genes required for integration, e.g. φSa1, νSaα, νSaβ, νSaγ, GIHunter does not perform well in identifying such islands. By contrast, JS-CB solely relies on codon usage bias for GI identification, and is, therefore, able to identify islands which might have lost their mobility or integration genes. However, the long-term resident islands such as νSaβ, which are likely to have their composition ameliorated to that of the host, were difficult to detect using JS-CB. Compared with JS-CB, PredictBias uses two criteria, namely dinucleotide bias and codon usage bias to delineate GIs; atypicality in either is considered a GI signature. Thus, clusters of ORFs showing atypical dinucleotide bias but not atypical codon usage bias were also annotated as GIs. Furthermore, JS-CB identifies atypical genes by assessing the similarities of the genes against each other rather than assessing the disparities against the genome background, thus identifying even clusters of weakly atypical genes. This may explain the better performance of JS-CB in comparison with the other methods of GI detection. Notably, JS-CB achieves overall highest accuracies by considering only codon usage bias as the discriminant criterion, outperforming the methods that use multiple criteria, such as PredictBias that uses pathogenicity-related gene information in addition to dinucleotide bias and codon usage bias to identify islands.

### 3.2. Comparison of JS-CB and GEMINI

We further compared JS-CB with a just-published genome mining tool, GEMINI, that performed well in delineating GIs in the Liverpool epidemic strain of *Pseudomonas aeruginosa* [21]. In application to the *P. aeruginosa* LESB58 genome, JS-CB identified wholly or partially all four verified islands at its default parameter setting (table 4). By contrast, GEMINI robustly identified three out of the four verified islands (VI-1, VI-2, VI-4) but missed completely VI-3 [21]. This level of performance was achieved by JS-CB by predicting 35.5% of the LESB58 genome as alien, while that by GEMINI by predicting approximately 13% of the genome as alien. When we readjusted the threshold of JS-CB (clustering threshold now set to 1E-28, see [12]) to obtain a conservative estimate of alien DNAs, same as that observed with GEMINI, JS-CB could still detect all four verified islands, with approximately 54% of the total nucleotides from the four verified islands classified as alien, while GEMINI classified approximately 84% of the nucleotides of the verified islands as alien. Both JS-CB and GEMINI identified three of four verified islands at the 50% overlap cut-off. The overall higher sensitivity of GEMINI could be attributed to its segmentation approach that enables detection of large islands with high precision; VI-4 is a large GI with 101 genes, approximately 98% of this island was detected by GEMINI, while JS-CB could detect only approximately 44%. By contrast, the application of GEMINI to MRSA genomes revealed its inability to localize the SCC*mec* island in N315, Mu50 and COL strains. GEMINI could identify only approximately 500 nucleotides of the SCC*mec* island for USA300 and MW2 at its default parameter setting. GEMINI predicted approximately 7% of the genome as alien for the five MRSA strains (table 4). When we readjusted the algorithm parameters for GEMINI (segmentation threshold and two-step clustering thresholds now set to 1E-11, 1E-13 and 1E-4 respectively; the default thresholds were earlier set based on *P. aeruginosa* genome analysis, see [21]), so that GEMINI predicts a similar proportion of a MRSA genome as alien as does JS-CB (approx. 27%). GEMINI was still not able to robustly identify the SCC*mec* island in all strains; only approximately 23%, approximately 50%, approximately 76%, approximately 92% and approximately 26% of SCC*mec* were identified in USA300, MW2, N315, Mu50 and COL strains respectively, while these numbers were approximately 99%, approximately 99%, 100%, approximately 99%, approximately 51% for JS-CB (tables 1 and 4).

**Table 4.** Comparison of JS-CB and GEMINI: Performance of JS-CB and GEMINI in identifying four verified islands in *P. aeruginosa* LESB58 strain (*a*) and SCC*mec* island in five MRSA strains (*b*) respectively was assessed at the default parameter setting and at two other parameter settings (see text). The known and predicted GI coordinates in the genomes are shown in base pairs (bp).

(*a*)										
verified islands in *P. aeruginosa* LESB58	VI-1		VI-2		VI-3		VI-4			
	start	end	start	end	start	end	start	end		
verified islands co-ordinates	2 504 700	2 551 100	2 690 450	2 740 350	2 751 800	2 783 500	2 796 836	2 907 406		
JS-CB co-ordinates at default parameters (native cluster size 64.5%)	2 506 736	2 560 126	2 690 501	2 786 349	2 690 501	2 786 349	2 798 946	2 929 279		
JS-CB co-ordinates at altered parameter settings (native cluster size 87%)	2 525 630	2 560 126	2 708 796	2 737 803	2 756 461	2 786 349	2 858 302	2 906 455		

(*b*)										
**SCC***mec* island in MRSA strains	**USA300**		**MW2**		**N315**		**Mu50**		**COL**	
	**start**	**end**	**start**	**end**	**start**	**end**	**start**	**end**	**start**	**end**
SCC*mec* co-ordinates	34 173	57 914	34 150	58 278	34 158	87 119	34 158	87 085	34 173	68 085
GEMINI co-ordinates at default parameters (native cluster size approx. 92%)	52 486	53 052	52 959	53 531	—	—	—	—	—	—
GEMINI co-ordinates at altered parameter settings (native cluster size approx. 73%)	52 487	75 088	39 574	51 676	45 020	85 415	36 374	85 373	59 234	92 806

SCC*mec* is characterized by several ORFs with atypical codon usage and GC content at the third codon position [4,28,29]. As JS-CB clusters genes based on codon usage bias, it is better able to exploit codon-specific information and therefore was able to localize SCC*mec* in the MRSA genomes. By contrast, GEMINI identifies GIs independent of codon information, by examining higher order oligonucleotide compositional biases, and therefore it may miss islands with atypical codon usage biases that are not reflected as atypicality in oligonucleotide composition. GEMINI, by virtue of its ability to analyse multiple genes simultaneously through a recursive segmentation process, localizes large islands more efficiently, which may appear fragmented in the predictions by bottom-up, gene-by-gene analysis methods such as JS-CB that may misclassify weakly atypical genes or compositionally ambiguous genes. Our results from the application of JS-CB and GEMINI to MRSA genomes reveal the complementary strengths of these two methods, which provides a basis for further future research towards the integration of complementary methods for attaining still better accuracy in GI prediction. We also noted that the performance of the GI prediction methods varies genomewise, reinforcing the need to develop methodologies for exploiting the complementary strengths of the prediction methods. An integrative approach to GI detection holds the promise to raise the accuracy bar across all genomes.

### 3.3. Identification of novel islands in HA- and CA-MRSA strains

JS-CB was able to identify many of the known islands, namely SCC*mec,* ACME, νSaα, SaPI-5, φSa1, φSa2, φSa3, νSa4, νSa1, νSa3 and φCOL, in the five strains we studied (table 1). However, the compositionally similar islands, such as SCC*mec* and ACME, and φSa3 and νSa4 (electronic supplementary material, table S1), were identified as one combined island instead of two separate islands (table 1). JS-CB missed νSaβ island in N315 and MU50, and νSaγ island in all five strains we examined. This may likely be a consequence of the amelioration process [30], whereby an island loses its inherent evolutionary signatures, after being subjected to the mutational processes of the host genome, over the passage of time since its acquisition. As JS-CB performed comparably or outperformed the current methods in localizing known GIs in five MRSA strains, we applied JS-CB to completely sequenced 22 HA-MRSA and nine CA-MRSA genomes to decipher yet unknown GIs in the MRSA strains. We discuss below our analysis of the JS-CB's novel predictions for the five reference strains, followed by the analysis of novel predictions for the remaining 26 strains.

**Figure 2.**
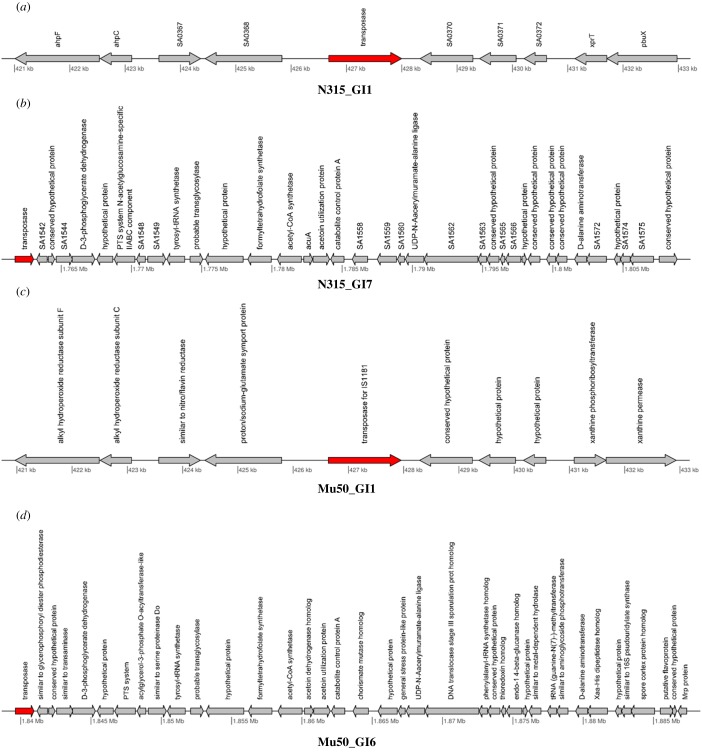
Gene maps of: (*a*) N315_GI1: transposase gene imparting mobility to the island has been highlighted in red; (*b*) N315_GI7: the presence of a transposase gene in the GI is indicated; (*c*) Mu50_GI1: the presence of a gene encoding transposase of insertion element is indicated; and (*d*) Mu50_GI6: the presence of a transposase gene and genes involved in metabolism in the GI is indicated.

**Figure 3.**
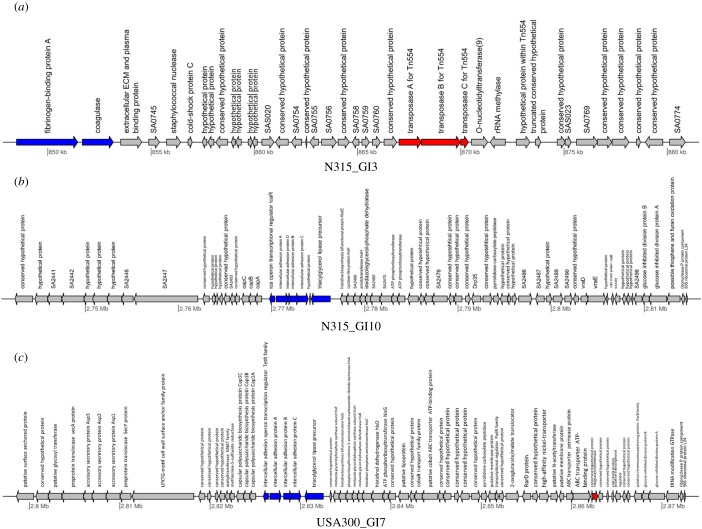
Gene map of: (*a*) N315_GI3: this GI possesses multiple copies of transposase genes (shown in red) and genes required for adhesion (i.e. virulence genes), namely fibrinogen-binding protein A and coagulase encoding genes (shown in blue); (*b*) N315_GI10: the presence of intercellular adhesin genes in the GI with a role in virulence is indicated; and (*c*) USA300_GI7: virulence genes (shown in blue) and a gene required for GI integration (shown in red) are indicated in this GI, suggesting a potential role in pathogenicity.

JS-CB's ability to more robustly identify the known islands in the MRSA strains motivated us to further explore and analyse the novel islands predicted by it. In the five strains that, we examined, JS-CB-identified 42 novel islands (electronic supplementary material, table S7). Several previous studies have enlisted features that typify GIs, e.g. atypical composition, presence of tRNA genes, direct repeats, integrase and transposase genes, and genes that may be imparting novel traits such as those encoding virulence or antibiotic resistance or other novel metabolic traits. To buttress our novel GI predictions, we collected further evidence in support of our predictions (electronic supplementary material, table S7). Of these, seven islands had either transposase or integrase genes, including N315_GI1, N315_GI7, Mu50_GI1 and Mu50_GI6 (figure 2*a*–*d*, electronic supplementary material, table S7). Fifteen islands harboured genes encoding virulence or antibiotic resistance factors (electronic supplementary material, table S7), including N315_GI3, N315_GI10 and US300_GI7 (figure 3*a*–*c*). Several islands included repeat regions, e.g. N315_GI1, N315_GI7, Mu50_GI1 and Mu50_GI6. While Mu50_GI1 has a repeat region in an internal site preceding a transposase gene, MU50_GI6 has repeat regions upstream of the island and at an internal site prior to a transposase gene. Likewise, N315_GI1 has a repeat region at an internal site before the transposase genes, while N315_GI7 has a repeat region just upstream of the island. The presence of transposase genes and repeat regions that are often associated with GI integration provides further evidence in support of our novel predictions. Some novel GIs predicted by JS-CB also harboured genes encoding virulence factors (electronic supplementary material, table S7). N315_GI3 (figure 3*a*) carries genes encoding fibrinogen-binding protein and coagulase, which were previously reported to be involved in virulence [31,32]; this island also harbours a transposase gene. While fibrinogen-binding protein helps *S. aureus* to colonize its hosts by facilitating attachment to a surface [33], coagulase, also a surface determinant involved in adherence, helps in converting fibrinogen to fibrin [34]. Both N315_GI10 (figure 3*b*) and USA300_GI7 (figure 3*c*) have genes encoding intracellular adhesion proteins. These proteins are critical in biofilm formation and adhering to surfaces [35]. USA300_GI7 also harboured an integrase/recombinase gene.

In addition to these five reference strains, we analysed novel predictions by JS-CB for the remaining 26 strains. The full list of putative GIs localized by JS-CB in the completely sequenced HA-MRSA and CA-MRSA genomes is given in the electronic supplementary material, table S8. JS-CB predicted 338 GIs across these 26 genomes. As little information is available about the locations of GIs including SCC*mec* in these strains, we first attempted to identify SCC*mec* in the 26 genomes. If a predicted island harboured SCC*mec* marker genes, namely the *mecA* and *ccr* genes, we annotated the predicted island as SCC*mec*. JS-CB could localize SCC*mec* in all 26 strains. The approximate location and size of these islands are conserved across all 26 strains, similar to the conservation observed in the five reference strains. If the marker genes were not found in the predicted islands, the sequence alignment program BLAST [36] was used to characterize the islands. Pairwise nucleotide sequence alignment of the predicted islands in 26 strains and the known islands (as in the five reference strains, table 1) was performed using BLAST. νSaα, νSaβ, φSa1, φSa2 and φSa3 were identified based on significantly high similarity of the predicted islands with these islands (query coverage greater than 60% and nucleotide identity greater than 80%). We thus identified 57 known islands across the 26 strains (electronic supplementary material, table S8). Among the remaining 281 predicted islands, 111 contain GI-specific features such as transposase, integrase or phage genes. Among these, 62 islands harbour either virulence or antibiotic resistance genes or both. To identify GIs shared among strains, we grouped the 281 GIs based on sequence similarity using CD-HIT [37]. GIs with high similarity (nucleotide identity greater than 80%) were grouped in a cluster. This yielded in total 22 clusters, each with five or more GIs (electronic supplementary material, table S8).

Several previous studies have reported GIs in MRSA strains [3,4,6,13,14], which were identified based on typical features of GIs, such as mobility genes, transposase, flanking tRNA genes which act as insertion sites, phage genes, insertion sequence elements and direct repeats flanking the GIs. However, with this approach we may miss GIs that lack these features yet have been mobilized through HGT or several long-time resident islands that may have lost some or all of such features. A comprehensive analysis of genomes for the presence of GIs thus requires a combination of complementary methods, including phylogenetic and composition-based or parametric methods. The novel MRSA GIs lacking typical GI-associated features therefore require further investigation.

### 3.4. Mosaicism of SSC*mec*

The SCC*mec* island carries a *mec* gene complex and chromosome recombinase (*ccr*) gene complex, and is integrated at integration site sequence (ISS) for SCC [38]. The *mec* gene complex includes *mecA* gene, its regulatory genes and insertion sequences [38]. The *ccr* gene complex includes *ccrA*, *ccB*, *ccrC* genes, and the flanking regions [38]. Depending on the allotype of the genes in the *mec* and *ccr* gene complexes, eight types of SCC*mec* islands have been identified. Of the five reference strains analysed, genes within the SCC*mec* island were segregated into two or more clusters by JS-CB (electronic supplementary material, table S9), revealing the mosaic structure of SCC*mec* and the likely distinct ancestries of the disparate segments composing the SCC*mec* island. Interestingly, methicillin resistance gene *mecA* and recombinase gene *ccr* (*ccrA, ccrB, ccrC*) were assigned to different clusters for N315 and Mu50 strains. GIs structurally similar to SCC*mec*, carrying *ccr* genes but lacking *mecA* gene have been reported previously in *Staphylococcus hominis* [39]; these islands were also shown to spontaneously excise [39], thus suggesting that the SCC*mec* like islands lacking *mecA* gene might have originated earlier and later acquired the *mecA* gene to form a functional SCC*mec* island. An alternative explanation for the absence of *mecA* genes in SCCmec like islands could be the loss of *mecA* genes from the original SCC*mec* islands, resulting in the SCC*mec* like islands lacking *mecA* in some *Staphylococcus* genomes. Mosaic islands were not observed in the strains USA300 and COL, but a bipartite SCC*mec* island was observed in MW2. Of the remaining 26 strains, mosaic SCC*mec* was observed in 14 strains. Six of these 14 strains with mosaic SCC*mec* had *mecA* and *ccr* genes assigned to different clusters. A tripartite structure of SCC*mec* was observed in eight strains, and bipartite, quadripartite and pentapartite structures were observed in two strains each in the remaining 26 strains (electronic supplementary material, table S9). The differential mosaicism of SCC*mec* as observed in our study of course needs further investigation.

## 4. Conclusion

A gene clustering-based method, JS-CB, outperformed several GI prediction methods in identifying GIs in MRSA genomes. Evidence gathered from the literature and sequence comparison supported many of the novel GIs predicted by JS-CB. The putative functional role of the GIs identified by JS-CB indicates the proclivity of *S. aureus* to acquire foreign DNAs to become multidrug-resistant or metabolically distinct organisms. JS-CB further revealed the mosaic structures of many GIs including the SCC*mec* island, which calls for further studies to understand their significance and plausible role in the adaptation of *S. aureus* to the changing environment. Our study also revealed the complementary strengths of the methods, e.g. JS-CB and GEMINI, which can be exploited in future studies to further improve GI identification in bacterial genomes.

## Supplementary Material

Supplementary Tables

## Data accessibility

The genome data used are available at NCBI ftp site (ftp://ftp.ncbi.nlm.nih.gov/) and the software programs used are publicly available from the program websites (JS-CB to be provided on request).

## References

[1] Herold BC, Immergluck LC, Maranan MC, Lauderdale DS, Gaskin RE, Boyle-Vavra S, Leitch CD, Daum RS 1998 Community-acquired methicillin-resistant *Staphylococcus aureus* in children with no identified predisposing risk. JAMA 279, 593–598. (doi:10.1001/jama.279.8.593) 9486753 10.1001/jama.279.8.593

[2] Dufour P, Gillet Y, Bes M, Lina G, Vandenesch F, Floret D, Etienne J, Richet H 2002 Community-acquired methicillin-resistant *Staphylococcus aureus* infections in France: emergence of a single clone that produces Panton-Valentine leukocidin. Clin. Infect. Dis. 35, 819–824. (doi:10.1086/342576) 12228818 10.1086/342576

[3] Holden MTG et al. 2004 Complete genomes of two clinical *Staphylococcus aureus* strains: evidence for the rapid evolution of virulence and drug resistance. Proc. Natl Acad. Sci. USA 101, 9786–9791. (doi:10.1073/pnas.0402521101) 15213324 10.1073/pnas.0402521101 PMC470752

[4] Baba T et al. 2002 Genome and virulence determinants of high virulence community-acquired MRSA. Lancet 359, 1819–1827. (doi:10.1016/S0140-6736(02)08713-5) 12044378 10.1016/s0140-6736(02)08713-5

[5] Hiramatsu K *et al* 2013 Genomic basis for methicillin resistance in *Staphylococcus aureus*. Infect. Chemother. 45, 117–136. (doi:10.3947/ic.2013.45.2.117) 24265961 10.3947/ic.2013.45.2.117 PMC3780952

[6] Holden MTG et al. 2010 Genome sequence of a recently emerged, highly transmissible, multi-antibiotic- and antiseptic-resistant variant of methicillin-resistant *Staphylococcus aureus*, sequence type 239 (TW). J. Bacteriol. 192, 888–892. (doi:10.1128/JB.01255-09) 19948800 10.1128/JB.01255-09 PMC2812470

[7] Centers for Disease Control and Prevention. 1999 Four pediatric deaths from community-acquired methicillin-resistant *Staphylococcus aureus*—Minnesota and North Dakota, 1997–1999. MMWR Morb. Mortal. Wkly Rep. 48, 707–710. 21033181

[8] Hiramatsu K 2001 Vancomycin-resistant *Staphylococcus aureus*: a new model of antibiotic resistance. Lancet Infect. Dis. 1, 147–155. (doi:10.1016/S1473-3099(01)00091-3) 11871491 10.1016/S1473-3099(01)00091-3

[9] Lan R, Reeves PR 2000 Intraspecies variation in bacterial genomes: the need for a species genome concept. Trends Microbiol. 8, 396–401. (doi:10.1016/S0966-842X(00)01791-1) 10989306 10.1016/s0966-842x(00)01791-1

[10] Everitt RG et al. 2014 Mobile elements drive recombination hotspots in the core genome of *Staphylococcus aureus*. Nat. Commun. 5, 3956 (doi:10.1038/ncomms4956) 24853639 10.1038/ncomms4956 PMC4036114

[11] Huang H, Flynn NM, King JH, Monchaud C, Morita M, Cohen SH 2006 Comparisons of community-associated methicillin-resistant *Staphylococcus aureus* (MRSA) and hospital-associated MSRA infections in Sacramento, California. J. Clin. Microbiol. 44, 2423–2427. (doi:10.1128/JCM.00254-06) 16825359 10.1128/JCM.00254-06 PMC1489486

[12] Azad RK, Lawrence JG 2007 Detecting laterally transferred genes: use of entropic clustering methods and genome position. Nucleic Acids Res. 35, 4629–4639. (doi:10.1093/nar/gkm204) 17591616 10.1093/nar/gkm204 PMC1950545

[13] Diep BA et al. 2006 Complete genome sequence of USA300, an epidemic clone of community-acquired meticillin-resistant *Staphylococcus aureus*. Lancet 367, 731–739. (doi:10.1016/S0140-6736(06)68231-7) 16517273 10.1016/S0140-6736(06)68231-7

[14] Gill SR et al. 2005 Insights on evolution of virulence and resistance from the complete genome analysis of an early methicillin-resistant *Staphylococcus aureus* strain and a biofilm-producing methicillin-resistant *Staphylococcus epidermidis* strain. J. Bacteriol. 187, 2426–2438. (doi:10.1128/JB.187.7.2426-2438.2005) 15774886 10.1128/JB.187.7.2426-2438.2005 PMC1065214

[15] Vernikos GS, Parkhill J 2006 Interpolated variable order motifs for identification of horizontally acquired DNA: revisiting the *Salmonella* pathogenicity islands. Bioinformatics 22, 2196–2203. (doi:10.1093/bioinformatics/btl369) 16837528 10.1093/bioinformatics/btl369

[16] Pundhir S, Vijayvargiya H, Kumar A 2008 PredictBias: a server for the identification of genomic and pathogenicity islands in prokaryotes. In Silico Biol. 8, 223–234. 19032158

[17] Ganesan H, Rakitianskaia AS, Davenport CF, Tummler B, Reva ON 2008 The SeqWord genome browser: an online tool for the identification and visualization of atypical regions of bacterial genomes through oligonucleotide usage. BMC Bioinformatics 9, 333 (doi:10.1186/1471-2105-9-333) 18687122 10.1186/1471-2105-9-333 PMC2528017

[18] Wei W, Gao F, Du M-Z, Hua H-L, Wang J, Guo F-B 2016 Zisland explorer: detect genomic islands by combining homogeneity and heterogeneity properties. Brief. Bioinform. 18, 357–366. (doi:10.1093/bib/bbw019) 10.1093/bib/bbw019 PMC5429010 26992782

[19] Che D, Wang H, Fazekas J, Chen B 2014 An accurate genomic island prediction method for sequenced bacterial and archaeal genomes. J. Proteomics Bioinform. 7, 214–221.

[20] de Brito DM, Maracaja-Coutinho V, de Farias ST, Batista LV, do Rêgo TG 2016 A novel method to predict genomic islands based on mean shift clustering algorithm. PLoS ONE 11, e0146352 (doi:10.1371/journal.pone.0146352) 26731657 10.1371/journal.pone.0146352 PMC4711805

[21] Jani M, Mathee K, Azad RK 2016 Identification of novel genomic islands in Liverpool epidemic strain of *Pseudomonas aeruginosa* using segmentation and clustering. Front. Microbiol. 7, 1210 (doi:10.3389/fmicb.2016.01210) 27536294 10.3389/fmicb.2016.01210 PMC4971588

[22] Lin J 1991 Divergence measures based on the Shannon entropy. IEEE Trans. Inf. Theory 37, 145–151. (doi:10.1109/18.61115)

[23] Grosse I, Bernaola-Galván P, Carpena P, Román-Roldán R, Oliver J, Stanley HE 2001 Analysis of symbolic sequences using the Jensen-Shannon divergence. Phys. Rev. E 65, 041905 (doi:10.1103/PhysRevE.65.041905) 10.1103/PhysRevE.65.041905 12005871

[24] Langille MGI, Brinkman FSL 2009 IslandViewer: an integrated interface for computational identification and visualization of genomic islands. Bioinformatics 25, 664–665. (doi:10.1093/bioinformatics/btp030) 19151094 10.1093/bioinformatics/btp030 PMC2647836

[25] Dhillon BK et al. 2015 IslandViewer 3: more flexible, interactive genomic island discovery, visualization and analysis. Nucleic Acids Res. 43, W104–W108. (doi:10.1093/nar/gkv401) 25916842 10.1093/nar/gkv401 PMC4489224

[26] Waack S, Keller O, Asper R, Brodag T, Damm C, Fricke WF, Surovcik K, Meinicke P, Merkl R 2006 Score-based prediction of genomic islands in prokaryotic genomes using hidden Markov models. BMC Bioinformatics 7, 142 (doi:10.1186/1471-2105-7-142) 16542435 10.1186/1471-2105-7-142 PMC1489950

[27] Stothard P, Wishart DS 2005 Circular genome visualization and exploration using CGView. Bioinformatics 21, 537–539. (doi:10.1093/bioinformatics/bti054) 15479716 10.1093/bioinformatics/bti054

[28] Hiramatsu K, Cui L, Kuroda M, Ito T 2001 The emergence and evolution of methicillin-resistant *Staphylococcus aureus*. Trends Microbiol. 9, 486–493. (doi:10.1016/S0966-842X(01)02175-8) 11597450 10.1016/s0966-842x(01)02175-8

[29] Kuroda M et al. 2001 Whole genome sequencing of methicillin-resistant *Staphylococcus aureus*. Lancet 357, 1225–1240. (doi:10.1016/S0140-6736(00)04403-2) 11418146 10.1016/s0140-6736(00)04403-2

[30] Lawrence JG, Ochman H 1997 Amelioration of bacterial genomes: rates of change and exchange. J. Mol. Evol. 44, 383–397. (doi:10.1007/PL00006158) 9089078 10.1007/pl00006158

[31] Foster TJ 2005 Immune evasion by staphylococci. Nat. Rev. Microbiol. 3, 948–958. (doi:10.1038/nrmicro1289) 16322743 10.1038/nrmicro1289

[32] Jonsson P, Lindberg M, Haraldsson I, Wadstrom T 1985 Virulence of *Staphylococcus aureus* in a mouse mastitis model: studies of alpha hemolysin, coagulase, and protein A as possible virulence determinants with protoplast fusion and gene cloning. Infect. Immun. 49, 765–769. 4040889 10.1128/iai.49.3.765-769.1985 PMC261269

[33] Foster TJ, Höök M 1998 Surface protein adhesins of *Staphylococcus aureus*. Trends Microbiol. 6, 484–488. (doi:10.1016/S0966-842X(98)01400-0) 10036727 10.1016/s0966-842x(98)01400-0

[34] Boden MK, Flock JI 1989 Fibrinogen-binding protein/clumping factor from *Staphylococcus aureus*. Infect. Immun. 57, 2358–2363. 2744851 10.1128/iai.57.8.2358-2363.1989 PMC313455

[35] Heilmann C, Schweitzer O, Gerke C, Vanittanakom N, Mack D, Götz F 1996 Molecular basis of intercellular adhesion in the biofilm-forming *Staphylococcus epidermidis*. Mol. Microbiol. 20, 1083–1091. (doi:10.1111/j.1365-2958.1996.tb02548.x) 8809760 10.1111/j.1365-2958.1996.tb02548.x

[36] Altschul SF, Gish W, Miller W, Myers EW, Lipman DJ 1990 Basic local alignment search tool. J. Mol. Biol. 215, 403–410. (doi:10.1016/S0022-2836(05)80360-2) 2231712 10.1016/S0022-2836(05)80360-2

[37] Huang Y, Niu B, Gao Y, Fu L, Li W 2010 CD-HIT Suite: a web server for clustering and comparing biological sequences. Bioinformatics 26, 680–682. (doi:10.1093/bioinformatics/btq003) 20053844 10.1093/bioinformatics/btq003 PMC2828112

[38] Elements IWG-SCC. 2009 Classification of staphylococcal cassette chromosome mec (SCCmec): guidelines for reporting novel SCCmec elements. Antimicrob. Agents Chemother. 53, 4961–4967. (doi:10.1128/AAC.00579-09) 19721075 10.1128/AAC.00579-09 PMC2786320

[39] Katayama Y, Takeuchi F, Ito T, Ma XX, Ui-Mizutani Y, Kobayashi I, Hiramatsu K 2003 Identification in methicillin-susceptible *Staphylococcus hominis* of an active primordial mobile genetic element for the *Staphylococcal Cassette Chromosome mec* of methicillin-resistant *Staphylococcus aureus*. J. Bacteriol. 185, 2711–2722. (doi:10.1128/JB.185.9.2711-2722.2003) 12700250 10.1128/JB.185.9.2711-2722.2003 PMC154413

